# Современные тенденции в лучевой диагностике опухолей надпочечников

**DOI:** 10.14341/probl13733

**Published:** 2026-03-07

**Authors:** Н. Г. Мокрышева, Н. В. Тарбаева

**Affiliations:** Национальный медицинский исследовательский центр эндокринологии им. академика И.И. ДедоваРоссия; Endocrinology Research CentreRussian Federation

**Keywords:** образование надпочечника, инциденталома, лучевая диагностика, компьютерная томография, радиомика, текстурный анализ, adrenal mass, incidentaloma, radiological diagnosis, computed tomography, radiomics, texture analysis

## Abstract

В редакционной статье рассматриваются современные тенденции и новые технологии в лучевой диагностике опухолей надпочечников, такие как двухэнергетическая компьютерная томография и методы текстурного анализа с использованием машинного обучения. Особое внимание уделяется дифференциальной диагностике образований надпочечников, включая дифференциацию на несколько классов. Представлены данные исследований, демонстрирующие высокую диагностическую эффективность данных подходов, а также подчеркивается важность междисциплинарного и персонализированного подхода в ведении пациентов. Материал отражает актуальные достижения и перспективы развития радиологических методов в эндокринологии и онкологии.

## Abstract (en)

The editorial examines modern trends and new technologies in the radiological diagnostics of adrenal tumors, such as dual-energy computed tomography and textural analysis methods utilizing machine learning. Particular attention is paid to the differential diagnosis of adrenal masses, including multi‑class differentiation. Research data demonstrating the high diagnostic efficacy of these approaches are presented, and the importance of an interdisciplinary and personalized approach in patient management is emphasized. The material reflects current achievements and future prospects for the development of radiological methods in endocrinology and oncology.

Объемные образования надпочечников представляют собой значимую клиническую проблему, актуальную на стыке эндокринологии, онкологии и лучевой диагностики. Выявление опухолей надпочечников продолжает расти, что обусловлено широкой доступностью и частым применением визуализирующих методов. Важно отметить, что индивидуальный клинический контекст, включающий возраст, сопутствующую патологию, онкологический анамнез и гормональный статус, требует персонализированного подхода в каждом конкретном случае. Однако первым и зачастую определяющим этапом в алгоритме ведения пациента становится именно радиологическая диагностика.

Долгое время для формирования показаний к оперативному вмешательству использовались 2 параметра: размер опухоли и скорость роста при динамическом наблюдении. Это приводило к большому числу необоснованных вмешательств при опухолях более 3–4 см и недооценке злокачественного потенциала образований малого размера, в том числе адренокортикального рака и метастатического поражения надпочечников. В настоящий момент основное внимание сконцентрировано на денситометрических критериях злокачественности опухоли и на количественных показателях трехфазной компьютерной томографии, что отражено в проекте клинических рекомендаций «Инциденталома надпочечника» [1].

Согласно рекомендациям Европейского общества эндокринологии от 2023 г. [2], в качестве первого метода визуализации инциденталом надпочечников рекомендуется бесконтрастная компьютерная томография (КТ) (рис. 1). Если при этом КТ-картина соответствует доброкачественному образованию (гомогенная структура и плотность ≤10), дальнейшая визуализация не требуется. Согласно систематическим обзорам и метаанализам, проведенным в 2016 [3] и в 2022 гг. [2], у 3723 пациентов без отягощенного онкоанамнеза и с подтвержденными доброкачественными образованиями надпочечников плотность этих образований на бесконтрастном КТ составляла ≤10 ед.Н.

**Figure:**
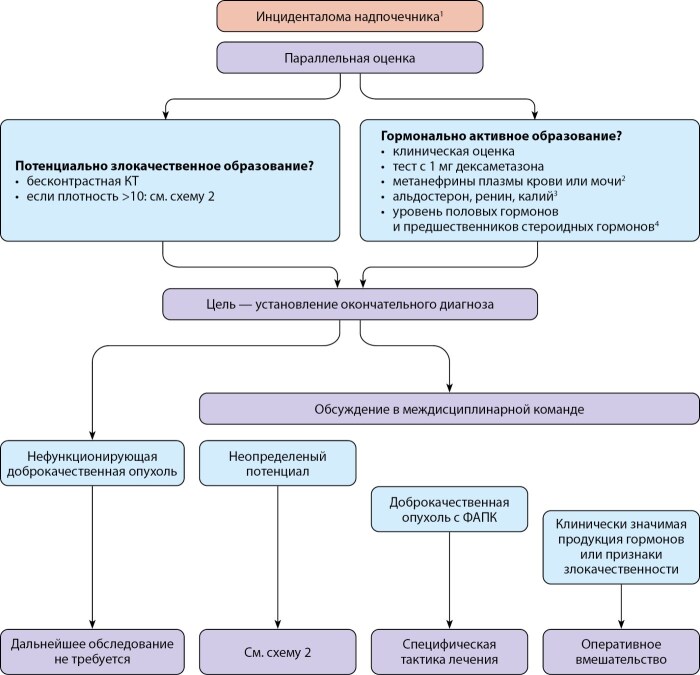
Рисунок 1. Блок-схема тактики ведения пациентов с инциденталомами надпочечников. *1) у небольшой подгруппы пациентов (образования надпочечников >4 см со злокачественными признаками, возраст <40 лет, беременность, выраженный избыток гормонов надпочечников) требуется срочная оценка; 2) необходимо только при опухолях надпочечников с плотностью >10 ед.Н; 3) только у пациентов с сопутствующей гипертонией и/или гипогликемией; 4) только у пациентов с клиническими, биохимическими или визуальными признаками, указывающими на адренокортикальный рак.

Основные сложности возникают при выявлении при бесконтрастной КТ образования надпочечника плотностью более 10 ед.Н (рис. 2). В таких случаях рекомендуется применение дополнительных методов визуализации и обсуждение в мультидисциплинарной команде с решением вопроса о необходимости оперативного вмешательства [2].

**Figure:**
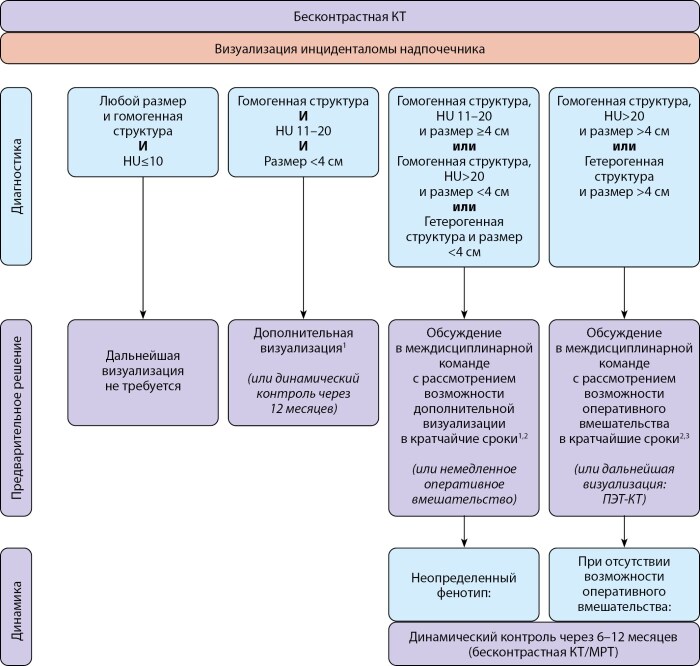
Рисунок 2. Визуализация инциденталом надпочечников. *1) дополнительная визуализация может варьировать в зависимости от возможностей медицинского учреждения, включая ФДГ-ПЭТ/КТ, МРТ надпочечников с химическим сдвигом или КТ с контрастным усилением; 2) в случае двусторонних поражений со сходными характеристиками, рассматривается выполнение биопсии; 3) после полного стадирования опухоли, включая КТ органов грудной клетки, брюшной полости и малого таза.

Неоднородность структуры значительного количества образований надпочечников остается одной из основных проблем диагностики, а вопрос о диагностической эффективности различных методов лучевой диагностики при гетерогенных опухолях остается открытым. В этих условиях особенно возрастает значимость разработки и внедрения новых методов визуализации, позволяющих неинвазивно и с высокой точностью определить морфологическую и функциональную природу опухоли до начала лечения. Современные подходы к радиологической диагностике образований надпочечников стремительно развиваются. Появляются новые критерии оценки, совершенствуются протоколы визуализации.

Отдельного внимания заслуживает внедрение двухэнергетической КТ (ДЭКТ), предоставляющей возможность получения виртуальных неконтрастных изображений, количественной оценки йодного контраста, а также создания карт плотности материалов. ДЭКТ представляет собой метод, при котором в результате использования двух типов рентгеновского излучения, различающимися по энергии, становится возможной дифференцировка тканей, различных по химическому составу, но со схожим коэффициентом поглощения [4]. Метод ДЭКТ обладает 96% чувствительностью и 100% специфичностью в дифференциальной диагностике образований надпочечников [5]. С помощью ДЭКТ появляется возможность декомпозиции изображения и разложения его на материалы, создания йодных карт, виртуальных неконтрастных изображений и монохроматических изображений на разных уровнях энергии [4].

В ФГБУ «НМИЦ эндокринологии им. академика И.И. Дедова» МЗ России проводятся исследования по оценке эффективности ДЭКТ в дифференциальной диагностике опухолей надпочечников различной степени злокачественности и возможности снижения лучевой нагрузки за счет применения виртуальных нативных изображений [6][7]. Полученные данные демонстрируют отсутствие статистически значимой разницы между виртуальными нативными изображениями и реальными нативными изображениями, а показатели ДЭКТ характеризуются чувствительностью 100%, специфичностью 81% в дифференциальной диагностике адренокортикального рака от аденом с низким содержанием жира.

Радиомика и машинное обучение открывают дополнительные горизонты для диагностики: анализ текстуры, формы и гетерогенности опухолей может позволить создавать предиктивные модели, способные повысить диагностическую точность и стандартизировать интерпретацию изображений [8][9]. С 2018 г. по настоящее время было опубликовано лишь несколько статей, касающихся текстурного анализа у пациентов с поражениями надпочечников. Тем не менее имеющийся мировой опыт применения текстурного анализа [10] показывает значимый потенциал в повышении точности диагностики с использованием количественных параметров медицинских изображений для анализа структурных особенностей тканей и актуальность проведения дальнейших исследований.

Результаты исследования Filippo Crimì и соавт. [11] показывают, что текстурный анализ имеет высокую точность в дифференциации доброкачественных и злокачественных поражений надпочечников (объединенная AUC 0,85), в частности, в дифференциации между образованиями коркового слоя надпочечников (аденомами и АКР).

В систематическом обзоре [12] проведен метаанализ 9 исследований по дифференциальной диагностике доброкачественных и злокачественных опухолей надпочечников, были получены средние значения метрик качества: чувствительность 0,80 (0,68–0,88), специфичность 0,83 (0,73–0,90).

Следует отметить, что практически все исследования в этой области сосредоточены на бинарной классификации (доброкачественные и злокачественные) или узких подгруппах (например, дифференцировка аденом на типы). При этом комплексные модели для диагностики одновременно нескольких классов новообразований надпочечников не разрабатывали. В доступной литературе только исследование [13] предлагает классификацию опухолей надпочечников с разделением их более чем на два типа. В то же время подход с многоклассовой классификацией представляется принципиально важным, поскольку феохромоцитомы, несмотря на потенциально злокачественный характер, требуют отличной от АКР и аденом диагностической и терапевтической стратегии [2].

В ФГБУ «НМИЦ эндокринологии им. академика И.И. Дедова» МЗ России в настоящее время проводится работа по определению истинной ценности машинного обучения в радиомике надпочечников [14][15][16].

Полученные результаты демонстрируют, что модели на основе текстурных признаков изображений КТ обеспечивает высокую диагностическую эффективность в дифференциальной диагностике доброкачественных и злокачественных опухолей надпочечников (AUC 0,954), а также в дифференциации феохромоцитом и аденом (чувствительность 89%). В то же время отмечены неудовлетворительные результаты при различении адренокортикального рака и феохромоцитом. Это может быть связано с рядом факторов, в том числе с гетерогенностью адренокортикального рака, который, как известно, обладает высокой структурной и функциональной неоднородностью [17]. По результатам исследования было показано, что существуют текстурные признаки с дискриминативной способностью для различения морфологических подтипов адренокортикального рака (low-grade и high-grade) со средним значением ROC-AUC в пределах 0,709–0,769. Выявлены статистически значимые текстурные паттерны, ассоциированные с высоким индексом пролиферации Ki-67 (>10%), которые характеризуется чувствительностью 83,3%, специфичностью 77,8%. Это подтверждает потенциал радиомики в улучшении диагностических процессов.

Настоящий выпуск журнала посвящен современным возможностям и перспективам диагностических методов. Мы стремимся отразить как достижения, так и существующие вызовы, подчеркнуть необходимость междисциплинарного подхода и акцентировать внимание на персонализации диагностики. Уверены, что представленная в номере информация будет полезна специалистам различных профилей и послужит стимулом для дальнейших исследований в этой важной и стремительно развивающейся области медицины.

## Дополнительная информация

Источники финансирования. Работа выполнена по инициативе авторов без привлечения финансирования.

Конфликт интересов. Авторы декларируют отсутствие явных и потенциальных конфликтов интересов, связанных с содержанием настоящей статьи.

Участие авторов. Все авторы одобрили финальную версию статьи перед публикацией, выразили согласие нести ответственность за все аспекты работы, подразумевающую надлежащее изучение и решение вопросов, связанных с точностью или добросовестностью любой части работы.
